# Assessing the Role of Voluntary Self-Isolation in the Control of Pandemic Influenza Using a Household Epidemic Model

**DOI:** 10.3390/ijerph120809750

**Published:** 2015-08-18

**Authors:** Qingxia Zhang, Dingcheng Wang

**Affiliations:** 1 School of Mathematical Sciences, University of Electronic Science and Technology of China, No. 2006, Xiyuan Avenue, West Hi-Tech Zone, Chengdu 611731, China; E-Mail: zqx121981@126.com; 2 School of Sciences, Southwest Petroleum University, No.8, Xindu Avenue, Xindu District, Chengdu 610500, China

**Keywords:** mathematical modeling, pandemic influenza, voluntary self-isolation, antiviral prophylaxis, household reproduction number

## Abstract

In the absence of effective vaccines, antiviral drugs and personal protective measures, such as voluntary self-isolation, have been a part of preparedness plans for the next influenza pandemic. We used a household model to assess the effect of voluntary self-isolation on outbreak control when antiviral drugs are not provided sufficiently early. We found that the early initiation of voluntary self-isolation can overcome the negative effects caused by a delay in antiviral drug distribution when enough symptomatic individuals comply with home confinement at symptom onset. For example, for the baseline household reproduction number $R_{H0}=2.5$, if delays of one or two days occur between clinical symptom development and the start of antiviral prophylaxis, then compliance rates of q≥0.41 and q≥0.6, respectively, are required to achieve the same level of effectiveness as starting antiviral prophylaxis at symptom onset. When the time to beginning voluntary self-isolation after symptom onset increases from zero to two days, this strategy has a limited effect on reducing the transmission of influenza; therefore, this strategy should be implemented as soon as possible. In addition, the effect of voluntary self-isolation decreases substantially with the proportion of asymptomatic infections increasing.

## 1. Introduction

Influenza viruses are associated with high morbidity and mortality in humans and continue to be a major threat to public health [1]. For example, Dawood *et al.* estimated that the emergence of pandemic H1N1 in 2009 resulted in 200,000 respiratory deaths and 83,000 cardiovascular deaths worldwide [2].

Because influenza is an important global public health concern, the methods by which pandemic influenza could be contained are of widespread interest, and a variety of control measures have been implemented to contain the spread of influenza strains. Vaccination is the most widely available form of disease control, and vaccination is most effective at the start of an epidemic [3]; however, several months would be required to produce a vaccine against a novel influenza strain [4,5,6]. Before effective vaccines would become available, prevention measures would be limited to antiviral medications and to personal and societal hygienic measures.

Previous research has indicated that antiviral drugs can reduce the risk of becoming infected with currently-circulating influenza strains and can inhibit infectivity [7,8,9]. The effective use of antiviral drugs is a critical problem for influenza control. The measure of providing antiviral prophylaxis to the close contacts of influenza patients has been recommended by the World Health Organization as a principle of early aggressive measures to prevent pandemic influenza [10,11]. The United Kingdom specifically implemented the policy of dispensing antiviral drugs to infected persons and their close contacts between May and July 2009, and Pebody *et al.* suggested that this strategy was highly effective in reducing the incidence of secondary cases [12]. Using stochastic epidemic simulations, Longini *et al.* showed that targeted antiviral prophylaxis (*i.e.*, offering antiviral prophylaxis to the close contacts of suspected index influenza patients) was an effective control measure to contain pandemic influenza until vaccines became available [1]. In addition, the combination of targeted antiviral prophylaxis and other interventions has been successfully used to combat the spread of pandemic influenza [13,14,15,16,17,18]. Thus, in the present study, household-based antiviral prophylaxis is considered as a control measure. As performed by Becker and Wang [8], household-based antiviral prophylaxis was carried out by dispensing antiviral drugs to household members immediately after the first household case showed clinical symptoms.

Unfortunately, logistical constraints, such as a limited distribution capacity and an insufficient stockpile, might limit the effect of antiviral drugs [4]. Because influenza is a highly contagious disease that can be transmitted via close contact with an infected individual, minimizing contact with infected people helps reduce transmission [19]. Intervention measures aimed at reducing the contact rates between infected and susceptible individuals should be considered. Voluntary home confinement of infected individuals (*i.e.*, voluntary self-isolation) can reduce contact between ill people and other community members; thus, voluntary self-isolation is usually considered as an intervention capable of limiting the transmission of pandemic influenza. The European Centre for Disease Prevention and Control (ECDC) also recommends this measure [20]. Because self-isolation restricts the activity of ill people, it is controversial [21]. The public’s doubts regarding the effectiveness of this intervention might also make it a difficult strategy to implement [21]. To ease these doubts, it is important to investigate the efficacy of voluntary self-isolation in the control of pandemic influenza. Mathematical models are powerful tools with which to study the dynamics of infectious diseases and to evaluate the effects of various control measures [22,23]. Moreover, because transmission within a household is the dominant mode of transmission of infections, household epidemic models have recently received widespread attention [7,13,24,25,26,27].

Moreover, certain studies have suggested that asymptomatic cases and asymptomatic infections indeed occur during influenza transmission. Based on active clinical follow-up and laboratory-confirmed outcomes, Papenburg *et al.* estimated that approximately 10% of A(H1N1) 2009 infections were completely asymptomatic [28]. Additionally, one recent study by Hayward *et al*. suggested that for the 2009 H1N1 pandemic, the proportion of asymptomatic individuals was as high as 70% to 80% [29]. Several earlier studies confirmed that asymptomatic infections also occurred in H5N1 pandemic influenza [30,31]. Note that the presence of asymptomatic infections likely affects the epidemic outbreak and the effectiveness of certain control measures. Hence, asymptomatic infection is a critical factor when considering the transmission dynamics of infectious diseases and pandemic control strategies. Many researchers have thus investigated the impact of asymptomatic cases and asymptomatic infections [1,15,32,33].

Given these considerations, we used a household epidemic model to investigate how household-based control measures, including household-based antiviral prophylaxis and voluntary self-isolation of symptomatic individuals within households, contribute to the containment of influenza outbreaks. We examined the effects of voluntary self-isolation alone and in combination with antiviral prophylaxis on the control of pandemic influenza. We also explored the impacts of a delay in implementing voluntary self-isolation and of asymptomatic infections on the effectiveness of voluntary self-isolation. “Self-isolation” means that symptomatic individuals stay and confine themselves at home [34]. In practice, it would be difficult for a government to offer antiviral drugs for prophylaxis, but not for treating patients. Therefore, as in [35,36], an antiviral prophylaxis strategy of treating symptomatic initial cases and offering prophylaxis to those who had close contact with these initial cases is considered. Hence, the term “antiviral prophylaxis” in this paper refers to the use of antiviral drugs in the treatment of the symptomatic index cases of influenza in a household and in the prophylaxis of those who have had close contact with these index individuals.

## 2. Methods

We considered the spread of an influenza strain within a community of households. A household refers to a group of people who share the same living facilities under a single shelter structure [37]. In general, people more often have contact with their household members than with other persons outside their households [21]. Suppose that the community consists of a large number of households of various sizes. Let $h_{n}$ denote the proportion of households of size *n* (n=1,2,⋯) in the community, and let $g_{j}=\frac{jh_{j}}{\sum_{n=1}^{\infty }nh_{n}}\;\left(j=1,2,\cdots \right)$ denote the probability that a randomly-selected community member resides in a household of size *j*.

Based on certain literature on epidemic modeling [37,38], we assume that after the disease is introduced into a household, the chance that a household member will be infected by infectious people outside the household is negligible relative to the chance that he or she will be infected by an infectious household member. In other words, outbreaks within affected households evolve independently of each other [8,37]. The assumption of independence between household outbreaks is likely questionable, but fortunately, this problem has been resolved by Ball *et al.* [39]. These researchers considered a model that explicitly allows disease transmission between households and showed that given a major outbreak, household outbreaks are actually approximately (*i.e.*, asymptotically) independent if the number of households is large [40]. The chain of infection in a household outbreak is denoted by $C=(c_{1},\;c_{2},\;c_{3},\;\cdots )$, where $c_{j}$ represents the number of infected individuals in the *j*-th generation. In this study, the primary household case is considered to be the first generation. Suppose only one introductory case lives in every infected household; hence, $c_{1}$ equals one, and $c_{2}$ is the number of individuals infected by the primary case in the same household. For any j≥2, $c_{j}$ represents the number of infected individuals infected by the previous generation. For example, considering a household of size 5, the members are called “a”, “b”, “c”, “d” and “e”. Suppose that this household consists of four susceptible individuals and one introductory case and that “a” is the introductory case and infects “b”, “c” and “d”, after which “b” infects “e”. Here, the chain is denoted by 1→3→1, i.e., $c_{1}=1,\;c_{2}=3,\;c_{3}=1$, $c_{i}=0\;\left(i\geq 4\right)$. The probability that an epidemic chain *C* occurs in a household of size *j* is denoted by P(C|j); $v_{H}$ denotes the average size of an outbreak within a household that is selected randomly from the community.

It is inevitable that infectious individuals infect susceptible persons outside their households. We assume that one *k*-th generation household case infects other susceptible persons outside his or her household according to a Poisson process with a rate of $\mu_{k}$ [41], which is the average number of infected persons generated by a single *k*-th generation infected individual. Additionally, the probability that one *k*-th generation case in a household outbreak infects *i* members outside his or her household is denoted by $\phi_{i,k}\;\left(i=0,\;1,\;2,\;\cdots ;\;k=1,\;2,\;\cdots \right)$. According to Ball *et al.* [39], under the condition that the number of households is large and the number of infected households is relatively small, the probability that a given infected household member will infect an individual outside his or her household who is residing in a previously-infected household is negligible compared to the probability that a given infected household member will infect an individual outside his or her household who is residing in a previously-uninfected household. That is, each individual infected by one *k*-th generation infective outside the latter’s household resides in an otherwise previously-uninfected household.

Let *q* denote the fraction of symptomatic individuals who comply with voluntary self-isolation. We assume that the voluntary home confinement of patients begins on the *l* day after symptom onset. As in [38], we assume that the infected individual’s symptoms appear $T_{I}$ days after infection. As infected persons can transmit the infection prior to the onset of their symptoms, even self-isolated individuals may transmit the infection outside their households. As above, we assume that a *k*-th generation infected individual creates other infected individuals outside his or her household according to a Poisson process with a rate of $\mu_{k}^{\prime }$, where $\mu_{k}^{\prime }$ is the mean number of cases that one *k*-th generation household patient infects outside of his or her household prior to voluntary self-isolation. Then, let $\phi_{i,k}^{\prime }\;\left(i=0,\;1,\;2,\;\cdots ;\;k=1,\;2,\;\cdots \right)$ represent the probability that one *k*-th generation household case infects *i* individuals outside his or her household before voluntary self-isolation.

During the voluntary self-isolation period of patients, we assume that the behavior of their household members is unconstrained. It is unrealistic to segregate infected individuals from their household members [42]; thus, we further assume that self-isolation does not have any impact on the contacts between the isolated individuals and their household members. That is, the transmission chain within a household is not affected by the voluntary self-isolation strategy.

In addition to symptomatic cases, infected individuals who do not develop clinical symptoms also play a major part in the transmission of influenza [43]. We therefore consider asymptomatic infections in our model. We assume that infected people with influenza develop clinical symptoms with a probability of *α*. We also assume that one *k*-th generation asymptomatic household case infects other susceptible persons outside his or her household according to a Poisson process with a rate of $\epsilon_{k}\mu_{k}$. The parameter $\epsilon_{k}\;\left(k=1,\;2,\;\cdots \right)$ is the reduction in the infectiousness of the *k*-th generation individuals with asymptomatic infection to other community members, where $0\leq \epsilon_{k}\leq 1$. The case $\epsilon_{k}=0$ represents the scenario in which asymptomatic infected people are not contagious, and $\epsilon_{k}=1$ corresponds to the scenario in which asymptomatic cases and symptomatic cases have the same infectiousness. The probability that one *k*-th generation asymptomatic case in a household outbreak infects *i* members outside his or her household is denoted by $\phi_{i,k}^{\prime \prime }\;\left(i=0,\;1,\;2,\;\cdots ;\;k=1,\;2,\;\cdots \right)$.

We assume that the epidemic is seeded by a single infected individual who arrives from another location. Here, *Y* denotes the total number of cases in which antiviral prophylaxis and voluntary self-isolation are implemented. The derivation method for the eventual mean number of infected individuals is based on the premise that each newly-infected individual in the community will start an independent epidemic process with the same eventual average number of patients. This method of determining the eventual mean number of infected individuals was used by Becker and Wang [8]. The eventual mean number of infected individuals, EY, can be obtained by:

(1) $$EY=\frac{v_{H}}{1-R_{H}},$$

where:

(2) $$\begin{array}{ccc} R_{H} & = & \alpha \sum_{j=1}^{\infty }g_{j}\sum_{C}P_{1}\left(C|j\right)\sum_{k=1}^{j}c_{k}\left(q\mu_{k}^{\prime }+\left(1-q\right)\mu_{k}\right)\\ & & +\left(1-\alpha \right)\sum_{j=1}^{\infty }g_{j}\sum_{C}P_{2}\left(C|j\right)\sum_{k=1}^{j}c_{k}\epsilon_{k}\mu_{k} \end{array}$$

which is the mean number of primary cases generated in the community by all of the infected individuals of an affected household that is selected randomly from the community [8]. This is also the mean number of households with infections that are generated by all infected individuals within a random household outbreak [8,14,44], where $P_{1}\left(C|j\right)$ corresponds to the probability of an infection chain within a household receiving antiviral drugs and $P_{2}\left(C|j\right)$ corresponds to the probability of an infection chain within a household not receiving antiviral drugs. We briefly outline the derivation and interpretation of Equation (1) in the Appendix. Obviously, the household reproduction number must be $R_{H}<1$ for Equation (1) to be valid.

To describe the effects of the control measures on the household reproductive number, $R_{H}$, as in [8], we adopt the approach of Glass and Becker [38] to describe within-household transmission. We outline the method of [38] as follows. Transmission within the household is based on the Reed–Frost model [37,45], but the probability of escaping being infected by a household case varies with the generation [8]. The level of infectiousness of infected individuals is measured by the size of the virus population carried by the individual. The size of the virus population follows a deterministic birth-death process, with birth rate *λ* and death rate *d*. In the absence of control measures, the virus population dynamics at first follow a deterministic birth process with a constant rate *λ*. $T_{I}$ days after infection, influenza virus particles are cleared at a rate *d* because the body’s immune system is activated. After antiviral drugs are dispensed to infected individuals, the effectiveness of these drugs is represented by an additional death rate, *δ*, in the virus population. When antiviral drugs are dispensed to susceptible individuals, the protective effects of these drugs are reflected in the reduction of the per contact probability of transmission by a factor of *σ* [8]. In other words, the effects of antiviral drugs on susceptibility change the parameter *θ* to $\theta^{\sigma }$, where *θ* is the probability that a susceptible individual escapes infection by a single household member in the absence of antiviral drugs. For a full description of this change, please refer to [37]. According to [37], the parameter *θ* can be expressed by $\theta =\exp(-\int_{0}^{\infty }\lambda_{x}dx)$, where $\lambda_{x}$ represents the infectiousness function.

As mentioned above, because generations differ in the amounts of time between being infected and taking antiviral drugs, the probability that a susceptible individual escapes infection by an infected household member is related to that infected household member’s generation. We let $\theta_{i}\;\left(i=1,\;2,\;\cdots \right)$ denote the probability that a susceptible household member avoids being infected by a single *i*-th generation case.

## 3. Results

The containment of the spread of a disease in a community consisting of households is indicated by a reduction in the household reproduction number, $R_{H}$, to below one. For the purpose of containing an outbreak, we show the effectiveness of various interventions strategies in reducing the household reproduction number, $R_{H}$. As in [8], we show the change in the household reproduction number, $R_{H}$, with respect to the parameters *μ*, which is the average number of cases that an infected individual generates outside his or her household, and *θ*, which is the probability that an individual escapes infection by an infectious household member during the latter’s entire infectious period, with the goal of describing the effects of interventions on transmission. These definitions of parameters *μ* and *θ* apply to an entirely susceptible community in the absence of any control measures. Because the parameter *μ* quantifies between-household transmission and the parameter *θ* quantifies within-household transmission, they are two important factors for determining the values of $R_{H0}$. With the coordinates of *μ* and *θ*, we can display the results with a wide range of values of $R_{H0}$. The distribution of household sizes was simulated to be consistent with Australian census data from 2001. For simplicity, households with only one person and those with more than six persons were not considered, and the percentages of households with 2, 3, 4, 5, and 6 people were 44%, 21%, 21%, 10%, and 4%, respectively [8,38]. The values of the model parameters are given in Table 1. These values are consistent with experimental data, as in [8,38].

**Table 1:** Values of the model parameters.

Parameter	Value	Description
*λ*	4	Birth rate of the virus population.
*d*	5	Death rate of the virus population due to the immune response.
*δ*	0.5	Additional death rate of the virus population due to antiviral drugs.
*σ*	0.5	The factor by which the probability of infection during a single contact is reduced for an individual who is taking antiviral drugs.
$T_{I}$	2	The number of days after infection after which clinical symptoms appear.

### 3.1. Antiviral Prophylaxis and Voluntary Self-Isolation

The effects of prophylaxis with antiviral drugs have been studied previously [8]; the authors noted that timely distribution of antiviral drugs can reduce the household reproduction number, $R_{H}$, significantly. However, because the distribution capacity is limited in practice [4], it would be difficult to dispense antiviral drugs to affected households immediately after primary cases develop symptoms. Therefore, we considered the combination of antiviral prophylaxis and voluntary self-isolation as the interventions that would contain the transmission of influenza. We primarily focused on the role of voluntary self-isolation when antiviral drugs cannot be dispensed in a timely manner. The delays of one or two days were considered between symptom development and antiviral drug distribution. Home confinement of symptomatic individuals began at clinical symptom onset. $T_{A}$ is the time at which antiviral drugs are dispensed to all household members relative to the onset of the primary case’s symptoms. To evaluate the effect of voluntary self-isolation, the following six scenarios were considered:

- Strategy 1: antiviral prophylaxis (antiviral drugs were distributed to all household members at the introductory case’s symptom onset; *i.e.*, $T_{A}=0,\;q=0$);
- Strategy 2: antiviral prophylaxis (antiviral drugs were distributed to all household members one day after the introductory case’s symptom onset; *i.e.*, $T_{A}=1,\;q=0$);
- Strategy 3: antiviral prophylaxis (antiviral drugs were distributed to all household members two days after the introductory case’s symptom onset; *i.e.*, $T_{A}=2,\;q=0$);
- Strategy 4: antiviral prophylaxis and voluntary self-isolation (antiviral drugs were distributed to all household members one day after the introductory case’s symptom onset, where the rate of self-isolation compliance was q=0.5; *i.e.*, $T_{A}=1,\;q=0.5,\;l=0$);
- Strategy 5: antiviral prophylaxis and voluntary self-isolation (antiviral drugs were distributed to all household members two days after the introductory case’s symptom onset, where the rate of self-isolation compliance was q=0.5; *i.e.*, $T_{A}=2,\;q=0.5,\;l=0$);
- Strategy 6: antiviral prophylaxis and voluntary self-isolation (antiviral drugs were distributed to all household members two days after the introductory case’s symptom onset, where the rate of self-isolation compliance was q=0.7; *i.e.*, $T_{A}=2,\;q=0.7,\;l=0$).

**Figure 1:**
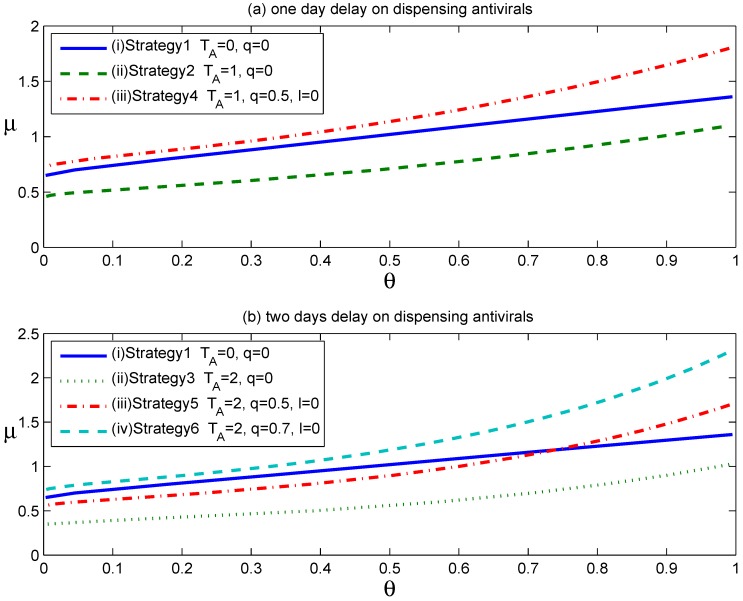
The effects of antiviral prophylaxis and voluntary self-isolation are displayed in two scenarios: (**a**) one day delay on dispensing antiviral drugs; (**b**) two days delay on dispensing antiviral drugs.

Figure 1 shows the effects of the above six strategies on reducing the household reproduction number, $R_{H}$, where α=1 and other parameters assume the values in Table 1. The curves in Figure 1 show the values of the parameter pairs (μ, θ) when $R_{H}$ equals one in the above six scenarios. For each curve in Figure 1, the parameter pairs (μ, θ) that satisfy $R_{H}>1$ lie above the $R_{H}=1$ curve, and those that satisfy $R_{H}<1$ lie below the $R_{H}=1$ curve.

As shown in Figure 1a, Curve (iii) lies above Curve (ii), implying that the strategy of confining patients at home expands the set of parameter values (μ, θ) for which $R_{H}<1$. In other words, the implementation of voluntary self-isolation expands the set of scenarios for which containment is achievable. In addition, Curve (iii) also lies slightly above Curve (i), denoting that Strategy 4 is nearly as effective as Strategy 1 with regard to reducing the household reproduction number, $R_{H}<1$. Thus, assuming that 50% of symptomatic individuals complied with home confinement at symptom onset, the voluntary self-isolation would overcome the negative effect caused by an antiviral drug distribution delay of one day.

Figure 1b shows that the implementation of voluntary self-isolation was also effective when a two-day delay occurred between symptom development and the start of antiviral prophylaxis. Importantly, however, a high-enough compliance rate is required to achieve the same level of effectiveness as the strategy of dispensing antiviral drugs to affected households at symptom onset. Table 2 specifically lists the needed compliance rates to achieve the same level of effectiveness as Strategy 1 or $R_{H}<1$, corresponding to delays of one or two days from the start of antiviral prophylaxis after clinical symptom onset. These calculations assumed the baseline household reproduction numbers of $R_{H0}=2.5$ and θ=0.5.

**Table 2:** The needed compliance rates to achieve the same level of effectiveness as Strategy 1 or $R_{H}<1$.

Delay in start of antiviral prophylaxis	The compliance rate	The effectiveness of interventions
1 day $\left(T_{A}=1\right)$	q=0.41	same as the effectiveness of Strategy 1
2 days $\left(T_{A}=2\right)$	q=0.6	same as the effectiveness of Strategy 1
1 day $\left(T_{A}=1\right)$	q≥0.23	$R_{H}<1$
2 days $\left(T_{A}=2\right)$	q≥0.47	$R_{H}<1$

### 3.2. Voluntary Self-Isolation

We evaluated the effectiveness of voluntary self-isolation (as a single intervention) and explored how the household reproduction number, $R_{H}$, varied with the changes to the compliance rate, *q*.

**Figure 2:**
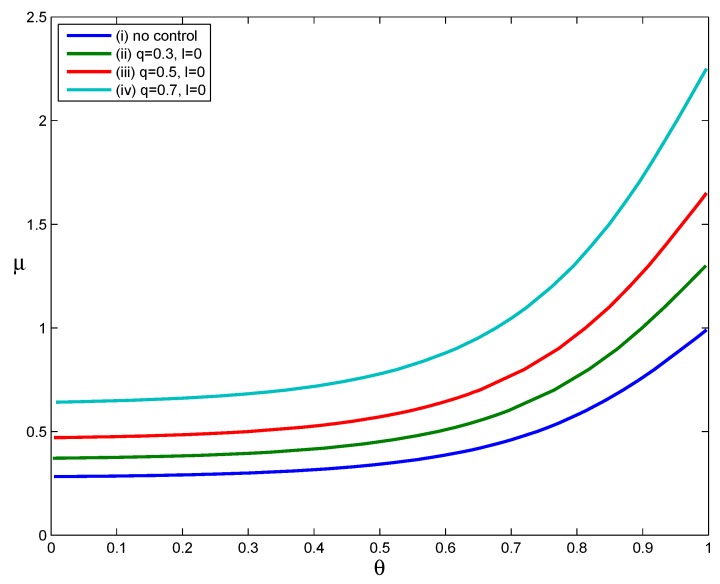
The effect of voluntary self-isolation.

The curves in Figure 2 show the values of parameters *μ* and *θ* when $R_{H}=1$ for scenarios in which (i) no interventions were implemented, (ii) the fraction of voluntary self-isolation was 0.3, (iii) the fraction of voluntary self-isolation was 0.5 or (iv) the fraction of voluntary self-isolation was 0.7. The last three scenarios assumed that infected individuals confined themselves to home at symptom onset (*i.e.*, l=0). The parameters α=1, δ=0, σ=1 and other parameters assume the values in Table 1. For each curve in Figure 2, the parameter pairs (μ, θ) that satisfy $R_{H}>1$ lie above the $R_{H}=1$ curve, and those that satisfy $R_{H}<1$ lie below the $R_{H}=1$ curve.

As Figure 2 shows, Curves (ii) through (iv) lie above Curve (i), implying that the implementation of voluntary self-isolation expands the set of parameter pairs (μ, θ) for which $R_{H}<1$. That is, the implementation of voluntary self-isolation effectively reduces the household reproduction number. Moreover, comparing the three Curves (ii), (iii) and (iv), we can see that the increase in the compliance rate *q* makes the $R_{H}=1$ curve shift upward and significantly expands the set of parameter points for which $R_{H}<1$. When we calculate values of $R_{H}$ using the parameter points (μ, θ) that lie on Curves (ii),(iii) and (iv), but suppose that no interventions are implemented, we obtain the values of $R_{H}$ in the intervals [1.3127, 1.3159], [1.6627, 1.6668] and [2.2673, 2.2729] corresponding to q=0.3, q=0.5 and q=0.7, respectively. As shown here, an intervention strategy based only on voluntary self-isolation can reduce values of $R_{H}$ from well above one to a value of one if a large proportion of infected individuals follow a public health department’s voluntary self-isolation guidelines. Clearly, as the compliance rate falls, the effectiveness of this strategy would be greatly reduced. However, if even 30% of cases are persuaded to stay at home at the onset of their symptoms, transmission can be reduced to some extent.

### 3.3. The Impact of Delay in Voluntary Self-Isolation

The above results were obtained under the assumption that infected individuals voluntary self-isolate at the onset of their symptoms. However, in practice, delays often occur between the onset of symptoms and the implementation of voluntary self-isolation. Therefore, we considered how a delay in the implementation of voluntary self-isolation affects the effect of the voluntary self-isolation strategy. Considering a situation in which the compliance rate is 0.5 (q=0.5) as an example, we examined the influence of a delay in the implementation of voluntary self-isolation on the household reproduction number, $R_{H}$.

**Figure 3:**
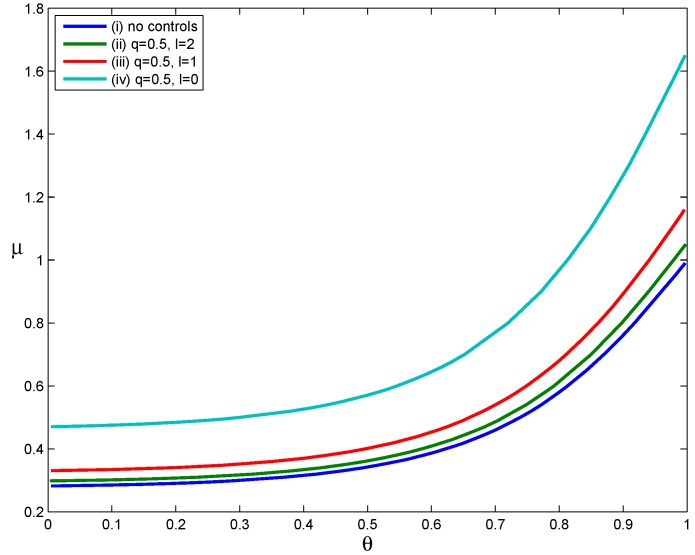
The impact of delay in voluntary self-isolation.

Four scenarios were used to evaluate the effect of delayed voluntary self-isolation (Figure 3): (i) no interventions, (ii) voluntary self-isolation beginning two days after symptom onset, (iii) voluntary self-isolation beginning one day after symptom onset and (iv) voluntary self-isolation beginning at symptom onset. The parameters α=1, δ=0, σ=1 and other parameters assume the values in Table 1. For each curve in Figure 3, $R_{H}<1$ when the parameter points (μ, θ) lie below the $R_{H}=1$ curve and $R_{H}>1$ when the parameter pairs (μ, θ) lie above the $R_{H}=1$ curve.

Comparing Curves (iv) and (i) in Figure 3, we can find that the implementation of voluntary self-isolation beginning at symptom onset significantly expands the set of parameter values (μ, θ) for which $R_{H}<1$. This suggests that implementing the voluntary self-isolation strategy as soon as symptoms appear leads to a significant expansion in the set of scenarios in which containment is achievable (relative to the scenario in which no control measures were implemented). However, as the time between symptom onset and the start of voluntary self-isolation increases, the set of scenarios in which containment is achievable becomes smaller. Therefore, the effectiveness of voluntary self-isolation in reducing transmission decreases when voluntary self-isolation is delayed. For example, Curve (ii) lies slightly above Curve (i), which implies that home confinement of symptomatic individuals beginning two days after the onset of symptoms results in a slight expansion in the set of scenarios in which containment is possible. In other words, voluntary self-isolation had little effect on mitigating the transmission of influenza when voluntary confinement of cases occurred two days after the onset of symptoms. Patients infected with influenza are infectious before their symptoms appear and are most infectious in the two to three days after symptom onset [8]. Therefore, voluntary self-isolation strategies are much more effective if implemented as soon as possible.

### 3.4. The Impact of Asymptomatic Infections

It is widely accepted that asymptomatic infection is an important route of influenza transmission [46]. Although asymptomatic cases can still shed the influenza virus, they are often excluded from the control objects, because they do not show apparent clinical symptoms. Therefore, the existence of asymptomatic infections will likely reduce the effectiveness of traditional control strategies. We examined the extent to which asymptomatic infections influence the effectiveness of voluntary self-isolation.

Due to their features, asymptomatic cases are difficult to diagnose, so clinical evidence of asymptomatic infection is extremely scarce [32]. The frequency of asymptomatic infections and the infectivity of asymptomatic individuals are thus hard to ascertain. Although there are a considerable variety of asymptomatic transmission scenarios, we assume that asymptomatic people have the same infectiousness as those with obvious clinical symptoms (*i.e.*, $\epsilon_{k}=1,\;k=1,\;2,\;\cdots$).

We assume that infected individuals would only consider placing themselves in self-isolation after showing symptoms; consequently, asymptomatic cases and those individuals who develop clinical symptoms, but are not willing to stay home, would infect the same number of people as they would in the complete absence of voluntary self-isolation measures.

Based on the assumptions above, considering only voluntary self-isolation, the household reproduction number, $R_{H}$, can be expressed by:

(3) $$\begin{array}{ccc} R_{H} & = & \alpha \sum_{j=1}^{\infty }g_{j}\sum_{C}P_{1}\left(C|j\right)\sum_{k=1}^{j}c_{k}\left(q\mu_{k}^{\prime }+\left(1-q\right)\mu_{k}\right)\\ & & +\left(1-\alpha \right)\sum_{j=1}^{\infty }g_{j}\sum_{C}P_{2}\left(C|j\right)\sum_{k=1}^{j}c_{k}\mu_{k} \end{array}$$

where *α* is the probability that an infected individual will develop symptoms.

As noted by Carrat *et al.* [47], the frequency with which infected individuals develop symptoms is a key consideration in intervention strategies. Some studies have suggested that about two-thirds of individuals infected with influenza exhibit clinical symptoms, and the remainder are asymptomatic [46,47]. According to Hayward *et al.* [29], asymptomatic individuals infected with seasonal and pandemic influenza comprise approximately three-fourths of all infected individuals; only one-fourth of infected individuals are symptomatic. Numerical simulations use three different values of *α* (α=1/4,2/3,1).

**Figure 4:**
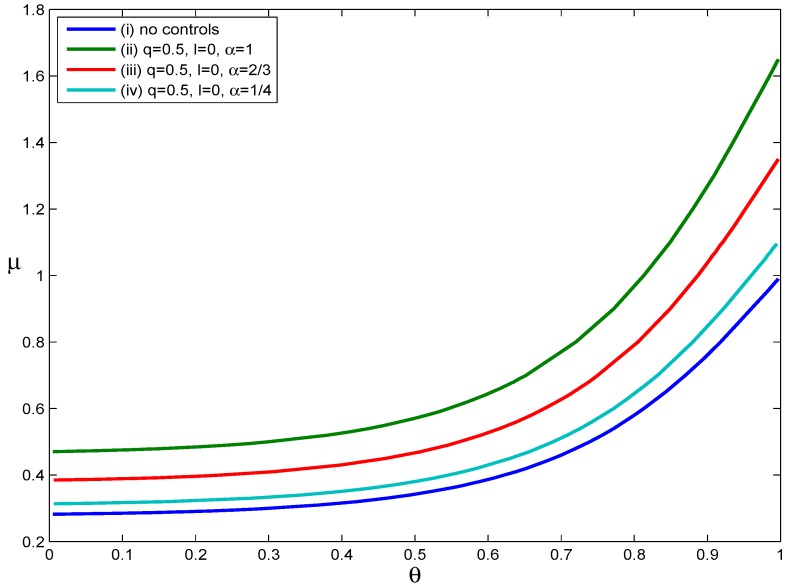
The impact of asymptomatic infected individuals.

Figure 4 illustrates how asymptomatic infections influence the effectiveness of voluntary self-isolation. Four scenarios were considered: (i) no intervention, (ii) q=0.5 and α=1, (iii) q=0.5 and α=2/3 and (iv) q=0.5 and α=1/4. For each curve in Figure 4, $R_{H}<1$ when the parameters (μ, θ) lie below the $R_{H}=1$ curve and $R_{H}>1$ when the parameters (μ, θ) lie above the $R_{H}=1$ curve. From Figure 4, we can see that as the value of the parameter *α* decreases, the $R_{H}=1$ curve moves down. This phenomenon implies that the decrease in the probability that an infected individual develops symptoms shrinks the set of scenarios in which containment is possible. In short, the effectiveness of voluntary self-isolation decreases as the probability of developing symptoms after infection decreases. For example, if an individual only has a one in four chance of developing symptoms after infection, voluntary self-isolation of symptomatic individuals with a compliance rate of q=0.5 did not substantially reduce disease transmission. Assuming no voluntary self-isolation, when the values of $R_{H}$ are calculated for the parameter pairs (μ, θ) on Curve (iv), $R_{H}$ values are approximately 1.11. From this, we can see that voluntary self-isolation has only a limited effect on reducing the values of $R_{H}$ if a high proportion of asymptomatic infections does indeed exist and if asymptomatic infected people have the same infectiousness as those with obvious clinical symptoms.

## 4. Discussion and Conclusions

In the absence of a sufficient quantity of vaccines, antiviral drugs are often considered an important countermeasure against the influenza virus.

The effects of targeted prophylactic use of antiviral drugs have been studied previously [8]; the authors of that study noted that administering antiviral drugs to affected households immediately after symptom onset in the initial case reduced transmission significantly; furthermore, the effectiveness of this strategy decreases as antiviral drug distribution time increases. However, a delay between distribution of antiviral drugs and onset of symptoms is usually inevitable in practice because of a limited capacity to quickly distribute drugs [4]. In this case, measures aimed at reducing the contact rates between ill and susceptible people should be considered. Therefore, voluntary self-isolation should be applied as an intervention to reduce the transmission of pandemic influenza when antiviral drugs cannot be dispensed in a timely manner.

Our results indicate that the implementation of a voluntary self-isolation strategy would improve transmission containment or, in other words, that the household reproduction number, $R_{H}$, would be reduced to less than one if a large proportion of symptomatic infected individuals complied with public health departments’ instructions to isolate themselves from other community members as soon as symptoms appeared. Naturally, if fewer infected individuals complied with this recommendation, this strategy would be less effective. However, if even a relatively small fraction of infected individuals were to comply with voluntary self-isolation, transmission could be reduced to some extent, and voluntary self-isolation is extremely critical when antiviral drugs are not immediately available. Importantly, the home confinement of infected individuals only succeeds when ill people are willing to comply with this containment measure. The effectiveness of voluntary self-isolation largely depends on public adherence to this intervention measure. With further understanding of pandemic influenza, the compliance with public health containment measures increased significantly [48]. Therefore, before possible intervention measures can be implemented against pandemic influenza, it might be necessary to disseminate knowledge of its clinical symptoms and associated containment measures to the public [34].

In addition, the efficacy of voluntary self-isolation is reduced if the implementation of voluntary self-isolation is delayed. Simulation results suggest that voluntary self-isolation has little impact on reducing the values of $R_{H}$ if voluntary self-isolation is implemented two days after the onset of symptoms. Therefore, one prerequisite for the voluntary self-isolation policy is the timeliness of its execution.

It is widely believed that asymptomatic infections are one of the major sources of influenza transmission. Here, we evaluated the impact of asymptomatic cases on the spread of influenza using the assumption that asymptomatic infected individuals were as infectious as symptomatic individuals [38]. We found that as the probability of infected individuals exhibiting symptoms decreases, the effectiveness of voluntary self-isolation likewise decreases. If the frequency of asymptomatic infections exceeds a given value, the effectiveness of voluntary self-isolation becomes very limited.

There are several requirements for the implementation of antiviral prophylaxis. (1) The stockpile of antiviral drugs must be adequate. (2) Infected individuals must develop clinically-recognizable symptoms and have access to healthcare. (3) Lastly, antiviral drugs must be dispensed rapidly to affected families. Some obstacles to the implementation of antiviral prophylaxis strategies may be found in practice because of the level of logistical support that would be required. Therefore, voluntary self-isolation should be implemented especially when antiviral drugs cannot be provided immediately. Unfortunately, voluntary self-isolation strategies may inconvenience individuals, lead to economic losses or even contribute to moral conflicts; thus, voluntary self-isolation remains a controversial strategy [21]. However, our results suggest that voluntary self-isolation is a feasible way to contain an influenza pandemic. It is worthwhile to note that voluntary self-isolation should be implemented as early as possible after symptoms develop and that, if an especially high proportion of cases are asymptomatic, other control measures should be considered, because the effectiveness of voluntary self-isolation will be reduced. These topics will be explored further in future studies.

## Appendix

Outline of the Derivation of Equation (1)

During the containment phase of the pandemic, as [8], we assume that each newly-infected person who is selected at random from the community gives rise to a new independent transmission process with the same eventual mean number of cases. Then, the eventual mean number of cases, EY, satisfies the following equation:

$$\begin{array}{cc} EY= & \alpha \sum_{j=1}^{\infty }g_{j}\sum_{C}P_{1}\left(C|j\right)\sum_{k=1}^{j}c_{k}\left(q\sum_{m=0}^{\infty }\phi_{m,k}^{\prime }\left(1+mEY\right)+\left(1-q\right)\sum_{m=0}^{\infty }\phi_{m,k}\left(1+mEY\right)\right)\\ & +\left(1-\alpha \right)\sum_{j=1}^{\infty }g_{j}\sum_{C}P_{2}\left(C|j\right)\sum_{k=1}^{j}c_{k}\sum_{m=0}^{\infty }\phi_{m,k}^{\prime \prime }\left(1+mEY\right). \end{array}$$

By simply computation, we obtain:

$$\begin{array}{cc} EY= & \alpha \sum_{j=1}^{\infty }g_{j}\sum_{C}P_{1}\left(C|j\right)\sum_{k=1}^{j}c_{k}+\left(1-\alpha \right)\sum_{j=1}^{\infty }g_{j}\sum_{C}P_{2}\left(C|j\right)\sum_{k=1}^{j}c_{k}\\ & +EY\left(\alpha \sum_{j=1}^{\infty }g_{j}\sum_{C}P_{1}\left(C|j\right)\sum_{k=1}^{j}c_{k}\left(q\mu_{k}^{\prime }+\left(1-q\right)\mu_{k}\right)+\left(1-\alpha \right)\sum_{j=1}^{\infty }g_{j}\sum_{C}P_{2}\left(C|j\right)\sum_{k=1}^{j}c_{k}\epsilon_{k}\mu_{k}\right). \end{array}$$

Denote:

$$v_{H}=\alpha \sum_{j=1}^{\infty }g_{j}\sum_{C}P_{1}\left(C|j\right)\sum_{k=1}^{j}c_{k}+\left(1-\alpha \right)\sum_{j=1}^{\infty }g_{j}\sum_{C}P_{2}\left(C|j\right)\sum_{k=1}^{j}c_{k},$$

which is the average size of an outbreak within a household that is selected randomly from the community, and:

$$R_{H}=\alpha \sum_{j=1}^{\infty }g_{j}\sum_{C}P_{1}\left(C|j\right)\sum_{k=1}^{j}c_{k}\left(q\mu_{k}^{\prime }+\left(1-q\right)\mu_{k}\right)+\left(1-\alpha \right)\sum_{j=1}^{\infty }g_{j}\sum_{C}P_{2}\left(C|j\right)\sum_{k=1}^{j}c_{k}\epsilon_{k}\mu_{k},$$

which is the mean number of primary cases generated in the community by all of the infected individuals of an affected household that is selected randomly from the community. Then, the eventual mean number of cases, EY, can be expressed as:

(1) $$EY=\frac{v_{H}}{1-R_{H}}.$$

Obviously, the household reproduction number must be $R_{H}<1$ for Equation (1) to be valid.
